# Functionalization of ethylene vinyl acetate with antimicrobial chlorhexidine hexametaphosphate nanoparticles

**DOI:** 10.2147/IJN.S65343

**Published:** 2014-08-27

**Authors:** Natalie J Wood, Sarah E Maddocks, Helena J Grady, Andrew M Collins, Michele E Barbour

**Affiliations:** 1 Oral Nanoscience, School of Oral and Dental Sciences, University of Bristol, UK; 2 Bristol Centre for Functional Nanomaterials, University of Bristol, UK; 3 Centre for Organised Matter Chemistry, School of Chemistry, University of Bristol, UK; 4 School of Health Sciences, Cardiff Metropolitan University, UK

**Keywords:** EVA, biomaterial, polymer

## Abstract

Ethylene vinyl acetate (EVA) is in widespread use as a polymeric biomaterial with diverse applications such as intravitreal devices, catheters, artificial organs, and mouthguards. Many biomaterials are inherently prone to bacterial colonization, as the human body is host to a vast array of microbes. This can lead to infection at the biomaterial’s site of implantation or application. In this study, EVA was coated with chlorhexidine (CHX) hexametaphosphate (HMP) nanoparticles (NPs) precipitated using two different reagent concentrations: CHX-HMP-5 (5 mM CHX and HMP) and CHX-HMP-0.5 (0.5 mM CHX and HMP). Data gathered using dynamic light scattering, transmission electron microscopy, and atomic force microscopy indicated that the NPs were polydisperse, ~40–80 nm in diameter, and aggregated in solution to form clusters of ~140–200 nm and some much larger aggregates of 4–5 μM. CHX-HMP-5 formed large deposits on the polymer surface discernible using scanning electron microscopy, whereas CHX-HMP-0.5 did not. Soluble CHX was released by CHX-HMP-5 NP-coated surfaces over the experimental period of 56 days. CHX-HMP-5 NPs prevented growth of methicillin-resistant *Staphylococcus aureus* when applied to the polymer surfaces, and also inhibited or prevented growth of *Pseudomonas aeruginosa* with greater efficacy when the NP suspension was not rinsed from the polymer surface, providing a greater NP coverage. This approach may provide a useful means to treat medical devices fabricated from EVA to render them resistant to colonization by pathogenic microorganisms.

## Introduction

Ethylene vinyl acetate (EVA) is in widespread use in biomedical and consumer materials. EVA and related polymers are used as a substrate for controlled drug release, for instance in intravitreal devices for the treatment of eye inflammation and disease;^1^ as an antibiotic-releasing coating for urethral catheters;^2^,^3^ and in artificial pancreases.^4^ EVA is used in intravaginal rings, which are devices used to deliver contraceptives^5^ and steroids, and are in development as means to deliver antiretroviral drugs.^6^,^7^ The mechanical properties of EVA are exploited in the fabrication of mouthguards^8^ and as cushioning material in the soles of sports shoes^9^ and posture-control shoes for individuals with health conditions affecting gait.^10^ It is inexpensive and easy to process, which has led to it being suggested as a replacement for other widely used biomedical materials such as silicones.^11^

Many of the applications of EVA are in environments where bacteria may be present, and bacterial colonization of EVA devices can lead to infection. Bacteria entering the eye during surgery can lead to endophthalmitis, a painful condition which may lead to partial or complete loss of vision; patients who received EVA implants have been reported to develop postsurgical infection by antibiotic-resistant *Staphylococcus aureus*^12^ as well as other infections including *Staphylococcus epidermidis*^13^ and unspecified gram-positive cocci.^13^,^14^ Catheter-associated urinary tract infections are common and represent a significant cause of morbidity. Commonly implicated bacteria in these infections include *Pseudomonas aeruginosa, S. epidermidis, Escherichia coli*, and *Enterococcus faecalis*.^15^ Mouthguards also become coated with bacteria during use, with many hundreds of species typically present in the mouth, both commensal and pathogenic. These can be persistent; after removing an EVA device from the mouth, some species were able to survive for a further 14 days.^16^ These may cause degradation of the device as well as generating an infection risk for other individuals coming into contact with the mouthguard.

The aim of this study was to functionalize EVA polymer with antimicrobial nanoparticles (NPs) which act as a slow release device for the broad-spectrum antimicrobial chlorhexidine (CHX).^17^ These NPs, first reported in 2013, have been shown to be effective against several pathogenic microorganisms when added in an aqueous colloid to bacterial culture, and absorb rapidly to surfaces such as glass and titanium,^17^ but they have not yet been investigated with respect to a biomedical polymer, nor in terms of antimicrobial efficacy when immobilized on a surface. The microorganisms investigated were selected owing to their significance to global health problems and difficulties encountered controlling them using traditional methods, and their association with biomaterials surfaces in vivo.

## Materials and methods

### Synthesis and characterization of NPs

Chlorhexidine hexametaphosphate (HMP) NPs were prepared by combining, at room temperature and pressure and under rapid stirring, CHX (as the digluconate salt in aqueous solution; Sigma-Aldrich Co., St Louis, MO, USA) and HMP (as the sodium salt in aqueous solution; Sigma-Aldrich) to effect final total concentrations of 5 and 5, or 0.5 and 0.5 mM of each. This process results in the formation of colloidal suspensions in which over 99% of the CHX is bound in NPs, with only 25 μM unbound remaining in solution.^17^ The resultant NPs will henceforth be referred to as CHX-HMP-5 and CHX-HMP-0.5.

CHX-HMP-5 and CHX-HMP-0.5 NP suspensions were freshly prepared and characterized for particle size and zeta potential by dynamic light scattering (DLS) at room temperature, using a Zetasizer Nano ZS (Malvern Instruments Ltd., Malvern, UK). All measurements were carried out on 700 μL of each NP suspension using DTS1061 disposable capillary cells (Zetasizer NanoZS; Malvern Instruments Ltd.). CHX-HMP-5 suspensions contained some sedimenting aggregates, so the specimen was allowed to settle before the supernatant was analyzed. Each numerical value reported is the average of the three measurements represented graphically; each measurement was carried out on a different NP suspension.

CHX-HMP-5 and CHX-HMP-0.5 NPs were deposited on carbon-coated copper grids (Agar Scientific Ltd., Essex, UK) and subjected to transmission electron microscopy (TEM; Jeol 120 kV 1,200 Mk2; Jeol, Tokyo, Japan). TEM grids were immersed in NP suspensions for 2 seconds, rinsed in deionized water for 2 seconds, and allowed to dry in air.

### Preparation and characterization of NP-functionalized materials

Specimens of medical grade EVA (Data Plastics, Witney, Oxfordshire, UK), measuring 7×7×3 mM, were coated with the NPs as follows: 200 mL of the colloidal suspension was prepared using freshly prepared reagents (to prevent hydrolysis of the HMP which may occur during lengthy storage). Each polymer specimen was cleaned by 10 minutes ultrasonication in industrial methylated spirits (IMS) and dried in air, then immersed in the rapidly stirred colloid for 30 seconds, removed and immersed in deionized water for 10 seconds to rinse, blotted using absorbent tissue to remove excess liquid, and allowed to dry in air.

The NP-functionalized specimens were examined using tapping mode atomic force microscopy (AFM; Nanoscope III; Digital Instruments, Santa Barbara, CA, USA). Specimens were subsequently coated with gold-palladium alloy using a sputter coating unit (Emitech SC7620; Quorum Technologies, Lewes, UK) and subjected to scanning electron microscopy (SEM; Phenom, Eindhoven, Netherlands).

### Soluble CHX elution from NP-functionalized materials

Eight specimens of EVA coated with CHX-HMP-5 and CHX-HMP-0.5 NPs were placed in individually labeled cuvettes suitable for ultraviolet spectrophotometry. Deionized water (2.5 mL) was added to the cuvettes, and they were sealed tightly using cuvette lids. These were agitated on an orbital shaker rotating at 150 rpm (SSM1; Bibby Scientific Limited, Staffordshire, UK). The cuvettes were kept sealed and were sampled for CHX concentration at intervals over a 56-day period as described elsewhere.^18^ Control sets were prepared where the specimens were immersed in deionized water and where they were immersed in a 25 μM CHX solution, which as mentioned above is the concentration of residual aqueous CHX in the CHX-HMP-5 colloidal suspension. CHX concentrations were calibrated with reference to standard solutions at 5–50 μM CHX and normalized to readings for water controls.

### Microbiology

Methicillin-resistant *Staphylococcus aureus* (MRSA; NCTC 13142, Global Resource Centre, Middlesex, UK) was cultured in Mueller–Hinton media. *Pseudomonas aeruginosa* NCIMB 8626 (ATCC 9027; National Collection of Type Cultures, Salisbury, UK) was cultured in nutrient broth. Both organisms were incubated aerobically at 37°C throughout the study.

Precultures of MRSA and *P. aeruginosa* were grown initially for 16 hours in appropriate media and equilibrated to optical density (OD) 0.1 (650 nm; SPECTROstar Nano; BMG LabTech, Ortenberg, Germany). Growth on polymer surfaces was determined by placing individual EVA specimens (n=3 per group) into the wells of a 24-well microtiter plate and adding 1 mL of liquid media (enough to cover the specimen). EVA specimens were cleaned by 10 minutes ultrasonication in IMS followed by either no treatment (control), 30 seconds immersion in stirred CHX-HMP-5 followed by 10 seconds in deionized water (CHX-HMP-5), or 30 seconds immersion in stirred CHX-HMP-5 without a rinse (CHX-HMP-5-H). Each well was inoculated with 10 μL of either the MRSA or *P. aeruginosa* precultures and then incubated for 24 hours at 37°C. Polymer specimens were removed from the wells using sterile forceps and transferred to sterile plastic Bijoux bottles (Fisher Scientific, Loughborough, UK) containing 1 mL phosphate-buffered saline (PBS). The tubes were vortexed for 1 minute to remove adherent bacteria, and cell suspension was serially diluted (10^−1^ to 10^−6^) in PBS and bacteria were enumerated using the Miles–Misra method.^19^

## Results

### Characterization of NPs

**Figure 1:**
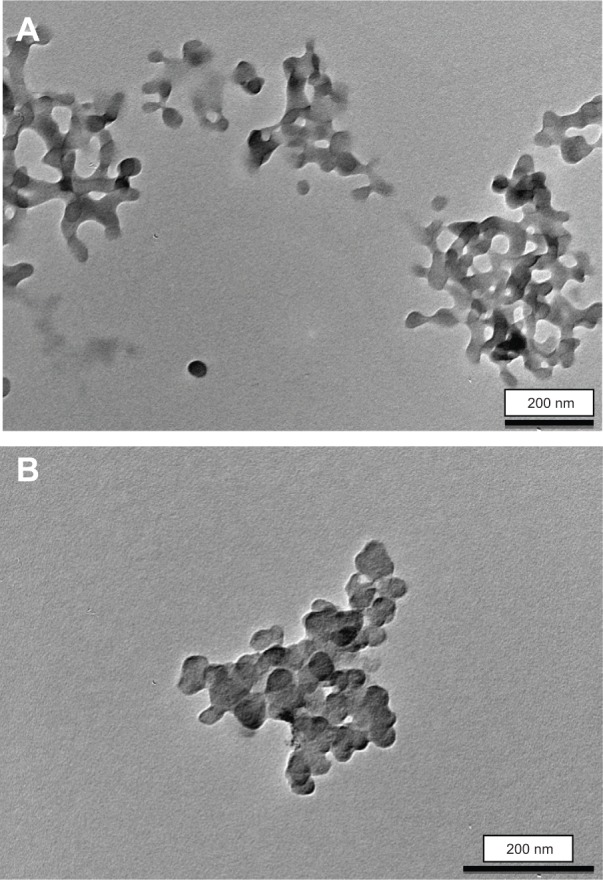
Transmission electron micrographs of CHX-HMP-5 (**A**) and CHX-HMP-0.5 (**B**) nanoparticles. **Note:** Scale bar 200 nm. **Abbreviations:** CHX, chlorhexidine; HMP, hexametaphosphate.

Transmission electron micrographs revealed the CHX-HMP-5 and CHX-HMP-0.5 NPs to be roughly spherical in shape with a typical diameter of ~40 nm (Figure 1). Individual NPs as well as small and large aggregates were observed, and the aggregates appeared in some cases to be composed of NPs fused together.

**Figure 2:**
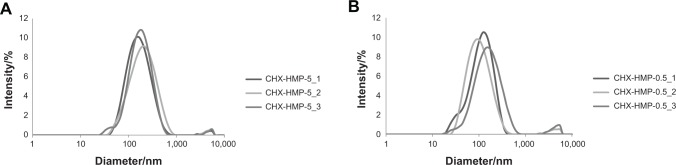
Dynamic light scattering data showing size distributions of CHX-HMP-5 (**A**) and CHX-HMP-0.5 (**B**) NPs, where CHX-HMP-X_1,2,3 indicate measurements in triplicate for concentration X. **Abbreviations:** CHX, chlorhexidine; HMP, hexametaphosphate; NPs, nanoparticles.

**Figure 3:**
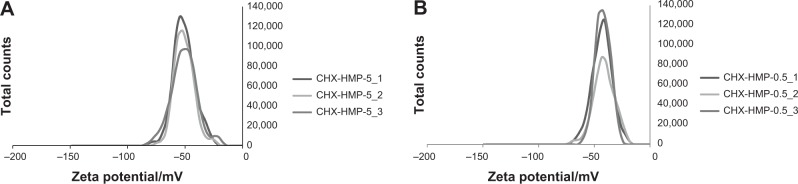
Zeta potential data showing charge distribution of CHX-HMP-5 (**A**) and CHX-HMP-0.5 (**B**) NPs where CHX-HMP-X_1,2,3 indicate measurements in triplicate for concentration X. **Abbreviations:** CHX, chlorhexidine; HMP, hexametaphosphate; NPs, nanoparticles.

DLS indicated that CHX-HMP-5 NP suspension was polydisperse with a large range of particle sizes, and showed two major peaks with a mean diameter of 202 nm (standard deviation (SD) 112 nm) and 4,670 nm (SD 790 nm). CHX-HMP-0.5 also showed significant polydispersity and two peaks with a mean diameter of 140 nm (SD 84 nm) and 4,210 nm (SD 998 nm) (Figure 2). For both suspensions there was also evidence of a population with smaller particle size evident as a “shoulder” in the region of ~40 nm. The zeta potential of CHX-HMP-5 NPs was −50.8 mV (SD 9.9 mV) and that of CHX-HMP-0.5 was −42.2 mV (SD 8.5 mV) (Figure 3).

**Figure 4:**
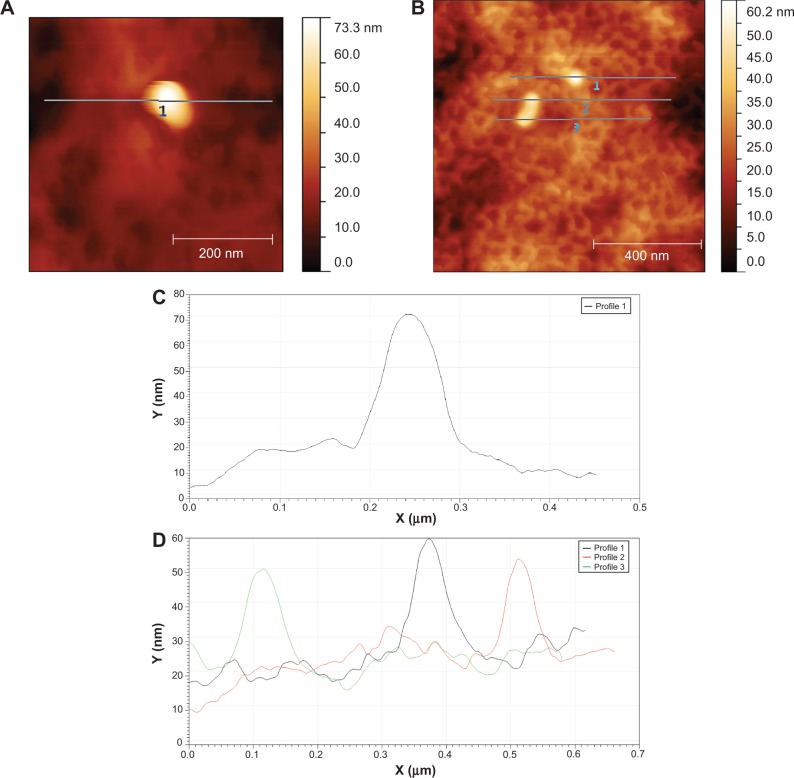
Atomic force microscopy images and line plots of a single CHX-HMP NP (**A**, **C**) and an aggregate of NPs (**B**, **D**). In (**D**), the black line corresponds to profile 1 in (**B**), the red line to profile 2, and the green line to profile 3, each showing a feature thought to be a nanoparticle or small aggregate. The textured background in (**B**) is thought to be the polymer surface. **Abbreviations:** CHX, chlorhexidine; HMP, hexametaphosphate; NP, nanoparticle.

**Figure 5:**
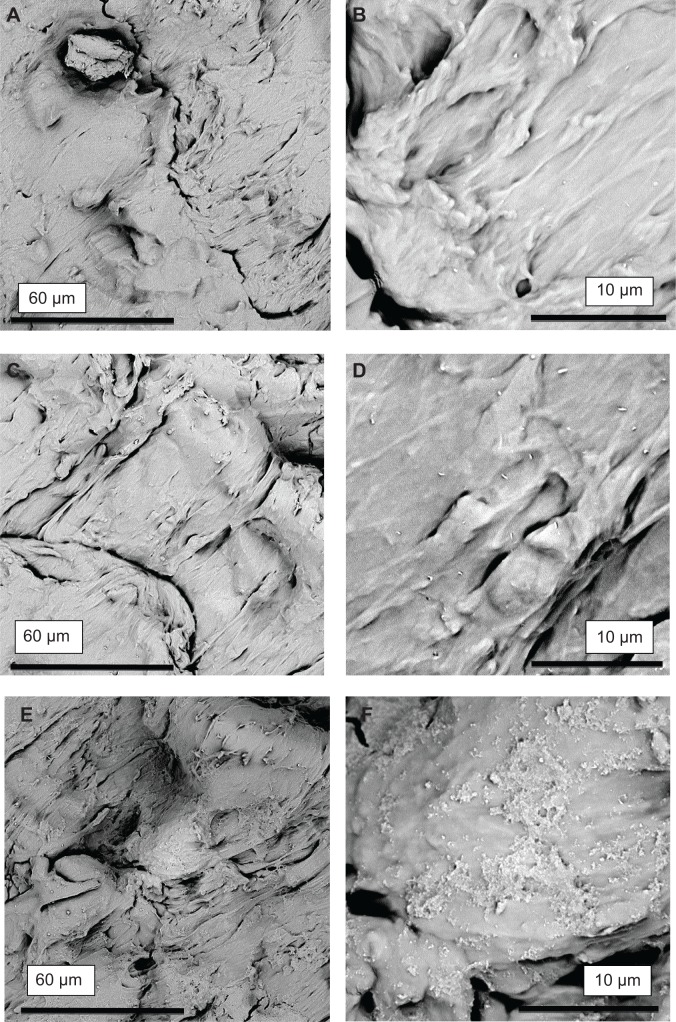
Scanning electron micrographs showing CHX-HMP nanoparticles on EVA polymer. (**A**, **B**) Control (no nanoparticles); (**C**, **D**) CHX-HMP-0.5 nanoparticles; (**E**, **F**) CHX-HMP-5 nanoparticles. The untreated EVA had a textured appearance (**A**, **B**). The CHX-HMP-0.5 surface was not clearly different from the control (**C**, **D**); there was no evidence of aggregations of nanoparticles as with the other material specimens. The CHX-HMP-5 surface displayed deposits of the porous aggregate coating much of the polymer surface (**E**, **F**). **Abbreviations:** CHX, chlorhexidine; HMP, hexametaphosphate; EVA, ethylene vinyl acetate.

Individual NPs and clusters were observed on EVA polymer surfaces using AFM (Figure 4) and SEM (Figure 5). Corresponding AFM line profiles extracted from Figure 4A and B showed single particles having a typical diameter 40–80 nm (Figure 4C and D). EVA polymer specimens functionalized using CHX-HMP-5 NPs showed aggregates of NPs on the polymer surface (Figure 5E and F). Those specimens functionalized using CHX-HMP-0.5 showed no obvious evidence of NP aggregates (Figure 5C and D); there were some small features but these were similar to features seen on the control, untreated surface (Figure 5A and B) and are thought likely to be inherent features of the polymer surface with any individual NPs too small to be resolved by this microscope.

### Soluble CHX elution from NP-functionalized materials

**Figure 6:**
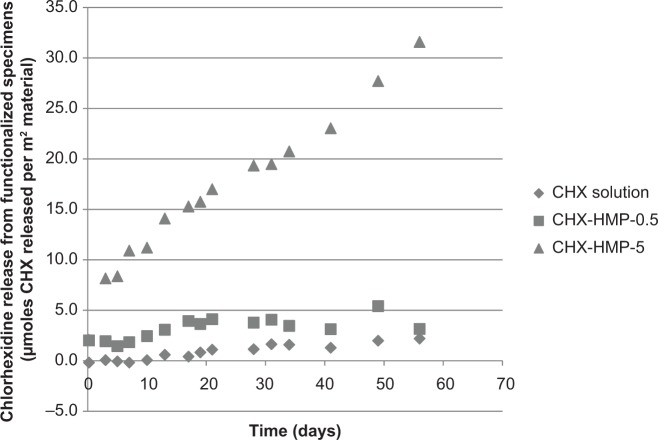
Chlorhexidine release from nanoparticle-functionalized EVA polymer expressed in μmoles CHX released per unit surface area of specimen as a function of time. The EVA functionalized with CHX-HMP-5 nanoparticles showed a sustained release of soluble CHX which was still ongoing at the end of the experimental period. There was a lower release from the CHX-HMP-0.5 specimens but this was almost all within the first day or two; there was little or no release after this time. The control specimens treated with 25 μM aqueous CHX solution did not show a release of CHX. **Abbreviations:** CHX, chlorhexidine; HMP, hexametaphosphate; EVA, ethylene vinyl acetate.

EVA polymer specimens coated with CHX-HMP-5 NPs released soluble CHX over an extended period (Figure 6). The CHX release continued at a constant rate for the duration of the experiment (56 days). The EVA specimens coated with CHX-HMP-0.5 NPs, by contrast, released little soluble CHX, and this release was only apparent immediately after the specimens were immersed in water. Specimens immersed in the 25 μM CHX solution showed little or no release of soluble CHX.

### Microbiology

For the uncoated, control EVA, *P. aeruginosa* recovered from the specimens were too numerous to count at all of the dilutions used; MRSA was recovered at 5×10^5^ cfu mL^−1^. For the EVA coated with a lower concentration of NPs (CHX-HMP-5), recovered bacteria were too numerous to count at all dilutions for *P. aeruginosa*, but no bacteria were recoverable for MRSA. For *P. aeruginosa*, the surrounding liquid medium was less turbid than the control (indicating less growth) despite high numbers of bacteria being recovered; for MRSA the surrounding medium was clear, supporting the surface observation of no growth. For the polymer pieces coated with a higher concentration of NPs (CHX-HMP-5-H), no bacteria were recoverable for either MRSA or *P. aeruginosa* and the surrounding media were clear in both instances.

## Discussion

The CHX-HMP NPs had indistinguishable size (~40 nm) and shape (approximately spherical) irrespective of the concentrations of reagents during precipitation, as indicated by TEM, whereas the particle sizes indicated by DLS were different for CHX-HMP-5 and CHX-HMP-0.5 (202 and 140 nm, respectively) and both were somewhat larger than that revealed by TEM, supporting the observation (Figure 1B) that the particles typically formed aggregates and that these aggregates were larger for the higher concentration suspension. Both NPs were negatively charged; CHX-HMP-5 had a larger net charge than CHX-HMP-0.5 (−50.8 and −42.2 mV, respectively).

CHX-HMP-5 NPs were readily observed as aggregates on EVA polymer specimens using SEM (Figure 5), and individual NPs could be seen using higher resolution AFM (Figure 4). No clear evidence of NP aggregates could be found on the CHX-HMP-0.5 surfaces; this, combined with the observation that the EVA CHX-HMP-0.5 specimens showed a very small and short-lived release of soluble CHX, suggests that there were few NPs on these surfaces. This is in contrast with other substrates such as glass, titanium, and alginate, which showed clear deposits of CHX-HMP-0.5 NPs following the same coating procedure.^17^ The mechanism of adhesion has not been fully elucidated, but is thought to be physical in nature owing to the observation that the NPs will aggregate and will adhere to most substrates irrespective of surface properties and functional groups.

The CHX-HMP-5 coated specimens showed a prolonged release of CHX over the 56-day period investigated here (Figure 5). Surfaces prepared in this way were effective against MRSA but not against *P. aeruginosa* growth on the material surface, although the growth medium showed evidence of a reduced microbial load. Immersing EVA in the same CHX-HMP-5 NPs and not subsequently rinsing the surface, yielding a higher coverage of NPs, prevented growth of MRSA and *P. aeruginosa*, both on the polymer surface and in the surrounding growth medium.

Owing to its importance as a biomedical and specifically oral polymer, there have been other attempts to confer CHX release onto EVA devices. CHX diacetate was incorporated into EVA and the resultant material showed reduced growth of *Streptococcus gordonii* and a modest effect on *Candida albicans*.^20^ Although the authors did not report CHX elution measurements per se, they did note that sequentially obtained eluates yielded successively lower antimicrobial activity, suggesting that the CHX elution reduced as a function of time. The antimicrobial activity was retained after the polymer sections were stored for 1 year, but they were stored dry, and it is not clear whether the effects would be repeated if the polymer was wet, as it would be in clinical use. In other studies, the same team incorporated CHX diacetate into EVA and did directly measure CHX release; they observed that it was released over a period of ~8 days.^21^,^22^

There have been other attempts to impart antimicrobial properties to EVA, such as the incorporation of essential oils into the polymer matrix.^23^ This approach resulted in films which reduced bacterial growth in a dose-responsive manner, but the oil release from these films had reached completion within 48 hours, in contrast to the more prolonged antimicrobial release achieved using the approach described here.

Urinary catheters represent such an infection risk that a number of antimicrobial catheters are available on the mass market. Although silver and nitrofural functionalized catheters were shown in a multicenter randomized clinical trial to offer little or no clinical benefit, at least for short-term (<14 days) catheterization,^24^ catheters coated with a CHX varnish have been reported,^25^–^27^ and the early laboratory and animal data are encouraging,^28^ showing a reduction in biofilm formation with the CHX-coated devices.

## Conclusion

An approach has been described to confer antimicrobial properties on EVA polymer by coating with CHX-HMP NPs. NPs of diameter ~40 nm and charge ~−50 mV were fabricated in an aqueous reaction using two reagents and the substrate was coated by immersion in the NP suspension. The two main advances which are demonstrated by this approach are: (1) The CHX release is sustained over a longer period than that achieved by other reported methods, and (2) the method to confer this CHX release is a simple dipping of the device into a suspension of the NPs, rather than incorporation during solvent-mediated polymer casting; thus, the CHX NP coating could be applied or replenished by the clinician, patient, or end user if required. This approach may find application in the developments of antimicrobial polymer-based medical devices, and in periodic topical applications of antimicrobial coatings for removable polymer products such as mouthguards.
